# Improvement of comorbid anxiety and depression in patients with migraine treated with injectable preventive calcitonin gene-related peptide antagonists: Review of clinical evidence

**DOI:** 10.1016/j.jsps.2024.101989

**Published:** 2024-02-15

**Authors:** Abubker Omaer, Abdulrazaq Albilali, Reem Bamogaddam, Fares Almutairi, Raghad Alsaif, Osama Almohammadi, Abdullah A Alhifany

**Affiliations:** a Clinical Pharmacy Department, King Saud Medical City, Riyadh, Saudi Arabia; b Neurology Unit, Department of Internal Medicine, College of Medicine, King Saud University, Riyadh, Saudi Arabia; c Pharmacy Department, King Fahad Armed Forces Hospital, Jeddah, Saudi Arabia; d Pharmacy Practices Department, College of Pharmacy, Umm Al-Qura University, Makkah, Saudi Arabia

**Keywords:** Eptinezumab, Erenumab, Galcanezumab, Fremanezumab, Depression, Anxiety

## Abstract

**Background:**

Migraine is often associated with depression and anxiety, leading to a diminished quality of life. Calcitonin gene‐related peptide (CGRP) antagonists have shown promise in treating migraines, but their effects on concurrent depression and anxiety have not been clarified.

**Methods:**

A literature review was conducted on ClinicalTrials.gov, PubMed, Ovid Medline, and EMBASE focusing on phase 3 clinical trials, post-hoc analysis studies, and real-world evidence (RWE) published in the past 5 years. The review primarily utilized patient-reported outcome tools, such as the Patient Health Questionnaire-9, Hamilton Depression Rating Scale, Beck Depression Inventory-II, generalized anxiety disorder (GAD)-7, and Hamilton Anxiety Rating Scale (HARS), to assess anxiety and depression in relation to CGRP-targeted monoclonal antibodies.

**Results:**

Out of 260 studies, 17 met the inclusion criteria. Eptinezumab lacked sufficient evidence regarding its impact on depression and anxiety. While sufficient evidence on its effect on comorbid anxiety was not available, fremanezumab was shown to significantly improve comorbid depression in one study while not achieving statistical significance in another. Erenumab and galcanezumab showed significant improvement in comorbid depression, implying possible benefits in patients with migraine. Galcanezumab showed faster relief from depressive symptoms than other injectable CGRP antagonists. Galcanezumab also exhibited improvements in GAD-7 scores for anxiety, although not statistically significant, whereas RWE showed promising HARS scores for both galcanezumab and erenumab.

**Conclusions:**

Galcanezumab and erenumab appear to be more effective in improving concurrent depressive and anxiety symptoms in migraine patients than fremanezumab. Notably, these psychometric questionnaires were not the primary outcome measures of the trials and were not specifically designed to investigate the effects of these medications on depression or anxiety. Further research is needed to fully understand the impact of CGRP antagonists on mental health disorders associated with migraines. These findings have implications for enhancing the overall well-being and quality of life in individuals with migraines and comorbid psychiatric conditions.

## 1 Background

Migraine is a complex brain disorder that affects approximately 12 % of the world’s population (Woldeamanuel and Cowan, 2017). Globally, it is the second most common cause of years lost due to disability (Vos et al., 2017). However, of the more than 25 % of migraine patients who could benefit from preventive therapy, only a small percentage receive it (Lipton et al., 2007).

Numerous studies have demonstrated that migraine is associated with a heightened risk of comorbid psychiatric disorders, particularly anxiety (Burch et al., 2019, Jette et al., 2008) and depression (Breslau et al., 1991), with the risk being more than double that in individuals without migraine (Jette et al., 2008, Buse et al., 2013, Fuller-Thomson et al., 2017). Irimia et al. found a positive linear correlation between monthly migraine days (MMDs) and the risk of anxiety and depression, with quality-of-life worsening when MMDs exceeded 12 (Irimia et al., 2021). The presence of comorbid anxiety and depression may also increase the risk of migraine progression over time (Guidetti et al., 1998) and lead to a poor response to both acute and preventive migraine treatments (Lantéri-Minet et al., 2005, Lucas et al., 2007, Schiano di Cola et al., 2019). Conversely, a study examining the overlap of mental illness, sleep disorders, and migraines/headaches in a large US organization from 2017 to 2021 revealed that individuals with both sleep and mental disorders, particularly depression and anxiety, are more prone to migraines, with 1.30 and 1.60 increase in the likelihood, respectively. Furthermore, those with only a sleep disorder, only mental illness, or both are respectively 1.33, 1.62, and 2.89 more likely to experience migraines (Merrill & Gibbons, 2023). Given the increased risk of comorbid psychiatric symptoms in migraine patients, it is essential to explore and evaluate the effectiveness of new migraine preventive treatments for those with comorbid anxiety and/or depression (Jette et al., 2008).

Calcitonin gene‐related peptide (CGRP) is a 37-amino-acid neuropeptide that has been strongly implicated in the pathophysiology of migraine (Ho et al., 2010). Small‐molecule CGRP receptor antagonists (gepants) and monoclonal antibodies (mAbs) to CGRP receptor have shown efficacy as CGRP‐targeted therapies for migraine (Ho et al., 2010, Ho et al., 2014, Petersen et al., 2005). Currently, four CGRP-targeted injectable mAbs (erenumab, galcanezumab, fremanezumab, and eptinezumab) are approved by the US Food and Drug Administration (FDA) for the preventive treatment of episodic and chronic migraine (Cohen et al., 2022). The results of pivotal phase 3 randomized clinical trials showed consistent efficacy and favorable tolerability of these CGRP-targeted mAbs compared with placebos for the preventive treatment of episodic and chronic migraine. Given the important association between uncontrolled migraine and comorbid mood disorders, post-hoc analyses and real-world evidence (RWE) studies have been conducted to assess the efficacy of the CGRP-target mAbs in patients with migraine with comorbid anxiety and depression (Lipton et al., 2020).

This study aimed to evaluate the available evidence regarding the benefit of using CGRP-targeted mAbs for treating patients with migraine with comorbid anxiety and depression to reveal possible correlations between improved comorbid anxiety and depression and improvements in terms of MMDs.

## 2 Methods

### 2.1 General methodology approach

We assessed the results of validated patient-reported outcomes (PROs) designed to detect depression and anxiety, which were collected during pivotal phase 3 randomized clinical trials of CGRP-targeted mAbs in patients with episodic or chronic migraine. Post-hoc analyses of these trials were also considered because some of the primary trials did not include assessments using such validated tools. RWE studies that reported the results of these measurements as secondary or exploratory outcomes were also included. The following were identified as the most commonly used PRO tools to assess depression or anxiety in clinical trials: Patient Health Questionnaire-9 (PHQ-9), Beck Depression Inventory-II (BDI-II), Hamilton Depression Rating Scale (HDRS), generalized anxiety disorder (GAD-7) scale, and Hamilton Anxiety Rating Scale (HARS). While the pivotal trials did gather data using these tools, it is imperative to mention that these were not the primary endpoints, and the studies were not specifically designed to assess the effects of injectable CGRP antagonists on depression or anxiety. All the studies referenced in this review were approved by the relevant Ethics Committee or Institutional Review Board approved (or waived) all the studies referenced in this paper.

### 2.2 PRO tools to assess depression

PHQ-9 is a diagnostic tool used to assess common mental health disorders, particularly depression (Kroenke et al., 2001). It follows the Diagnostic and Statistical Manual of Mental Disorders, Fourth Edition (DSM-IV) criteria, with scores ranging from 0 to 3 for each of the nine criteria and total scores indicating the severity of depression (mild, moderate, moderately severe, or severe) (Bell, 1994). The tool shows sensitivity and specificity of up to 88 % for diagnosing major depression. HDRS, used for over 50 years, is a primary standard to measure the severity of depressive symptoms in patients with various mental health disorders (Williams, 2001). It consists of 17 items, with higher total scores indicating more severe depressive symptoms, and the final score categorizes individuals into normal, or with mild, moderate, or severe depression (Bobo et al., 2016). BDI is a 21-item self-assessment tool commonly used to measure depressive symptoms based on DSM-IV criteria (Wang and Gorenstein, 2013). Each item has four response options on a 4-point scale, with a maximum score of 63 (García-Batista et al., 2018). The calculated score indicates depression severity, with scores ranging from normal (0–10) to extreme depression (scores > 40).

### 2.3 PRO tools to assess anxiety

GAD-7 is a validated tool used in primary care to detect GAD, with a cut-off score of 9 indicating accompanying anxiety (Spitzer et al., 2006). It has high sensitivity (up to 89 %) and specificity (82 %) for detecting GAD compared to structured psychiatric interviews. In migraineurs, a cut-off score of 5 indicated a sensitivity level of approximately 78.1 % and specificity of 74.6 % (Seo and Park, 2015a). HARS is a 14-item questionnaire originally used to assess anxiolytic efficacy in patients with anxiety neurosis, measuring both psychic and somatic anxiety symptoms (Maier et al., 1988). Responses range from 0 to 4, with a maximum score of 56. Scores below 17 indicate mild severity, 18–24 suggest mild to moderate severity, and scores above 25 indicate severe anxiety (Clark and Donovan, 1994).

### 2.4 Literature review strategy

**Fig. 1:**
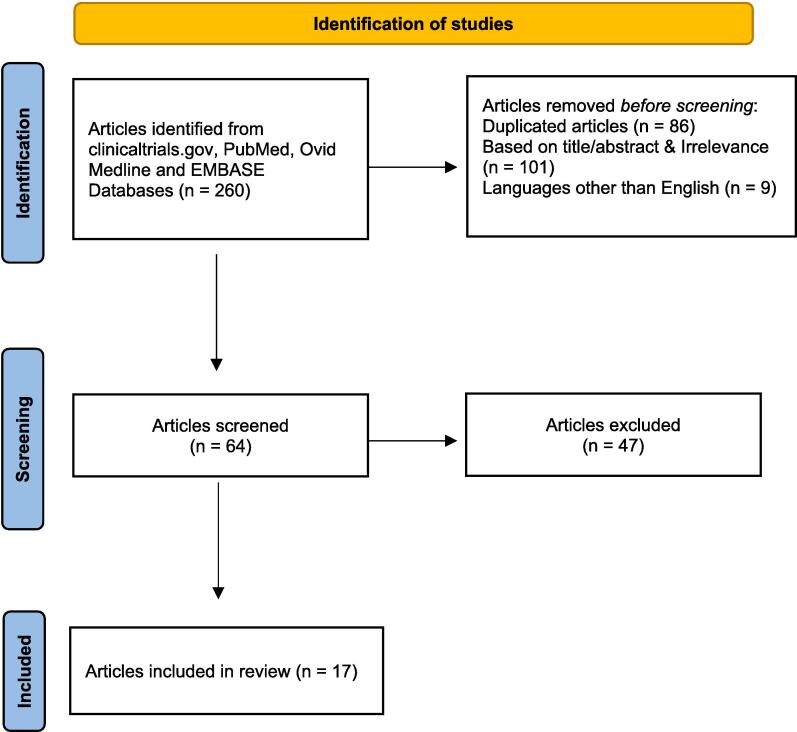
Flowchart summarizing the literature search strategy.

The databases searched in this review were ClinicalTrials.gov, PubMed, Ovid Medline, and EMBASE. The initial search results were narrowed down by applying the following criteria: randomized controlled trials, double-blind placebo-controlled trials, and articles published within the last decade. Original and review articles were used to extrapolate general information for the background of this review article. The MeSH terms used were: migraine disorders, depression, anxiety, calcitonin gene-related peptide, headache, and headache disorders. The search was limited to phase 3 clinical trials and post-hoc analyses published within the last 5 years. Duplicate articles or those published in languages other than English were excluded. Fig. 1 presents the flowchart summarizing the methodology used to prepare this literature review.

## 3 Results

Our initial search yielded 260 published studies, of which 64 were further screened for eligibility. We examined these articles to include evidence of the effect of injectable CGRP antagonists on anxiety and/or depression, using one of the tools described above. Only 17 articles complied with this requirement and results from these studies are summarized below.

### 3.1 Depression assessment using PHQ-9, HDRS, or BDI-II

#### 3.1.1 Eptinezumab

Assessment of depression using mean changes in PHQ-9, HDRS, or BDI-II scores was not included in the following studies: PROMISE-1 (Ashina et al., 2020), PROMISE-2 (Lipton et al., 2020), post-hoc analysis (Schim et al., 2022) of these two clinical trials, or the subsequent long-term study (Kudrow et al., 2021) exploring the safety and effectiveness of eptinezumab in patients with migraine.

#### 3.1.2 Erenumab

Neither the STRIVE (Goadsby et al., 2017), ARISE (Dodick et al., 2018) nor the pooled analysis (Lampl et al., 2022) of the five clinical trials examining the efficacy of erenumab in patients with migraine reported any PROs for depression and anxiety. However, an RWE that evaluated improvement in pain experience, medication use, and quality of life, including improvement in PHQ-9 score, in patients with chronic migraine resistant to botulinum toxin-A following erenumab treatment was informative. The study showed a significant improvement in PHQ-9 score, from a baseline score of 16.5 to 8.5 at 11 months (p = 0.001) (Talbot et al., 2021). Another RWE assessed the effect of erenumab in patients with chronic migraine with previous unsuccessful responses to preventive treatments. A significant improvement in depression was reported using HDRS and BDI-II, from baseline to after the sixth round of administration of erenumab (from 14.3 ± 0.9 to 10.5 ± 1.2 and from 17.0 ± 1.4 to 11.2 ± 1.6, respectively; p < 0.05) (Russo et al., 2020).

#### 3.1.3 Galcanezumab

The effect of galcanezumab on depression was not directly assessed in patients with episodic or chronic migraine in EVOLVE-1 (Stauffer et al., 2018), EVOLVE-2 (Skljarevski et al., 2018), REGAIN (Detke et al., 2018), or in the open-label extension (Pozo-Rosich et al., 2022) of phase 3 pivotal trials. However, the results from the secondary objectives of the CONQUER study (Maizels et al., 2020) that investigated galcanezumab therapy in adults with treatment-resistant migraine showed that the proportion of patients with minimal depressive symptoms (PHQ-9 score = 0–4)was increased significantly from 30.6 to 54.3 % (difference = 23.7 %), while the placebo group only reported a change from 28.7 to 42.3 % (difference = 13.6 %). Additionally, the percentage of patients with possible major depressive disorder (MDD) was decreased from 12.9 to 6.3 % with galcanezumab administration, and from 15.7 to 9.9 % with a placebo treatment. Patients treated with galcanezumab reported statistically larger mean decreases in PHQ-9 scores (−2.1 vs. − 1.2; p = 0.009) than those who received placebo.

Mean changes in HDRS or BDI-II scores were not identified in these primary clinical trials. However, a published multidimensional real-world outcome study on the effect of galcanezumab on total pain burden in migraineurs evaluated mean changes using these tools (Silvestro et al., 2022). The study showed that depressive symptoms, assessed using HDRS and BDI-II, were significantly improved from baseline to after the third round of administration (from 15 ± 10 to 10 ± 7.25 and from 13.5 ± 15 to 8 ± 10, respectively; p = 0.01). Further reduction was observed after the sixth round of administration (to 10 ± 7 and 8 ± 10.5, respectively; p < 0.003) (Silvestro et al., 2022).

#### 3.1.4 Fremanezumab

In a post-hoc analysis of the HALO-CM trial, changes from baseline in PHQ-9 scores during a 12-week fremanezumab treatment period were analyzed in a subgroup of patients who experienced moderate to severe depression (i.e., a score ≥ 10 on PHQ-9) at baseline (Lipton et al., 2021). This subpopulation showed reductions in mean PHQ-9 scores from baseline to week 12 with quarterly (−10.9 ± 1.01 points; reduction of 77.3 %) or monthly (−9.8 ± 0.93 points; reduction of 66.4 %) fremanezumab use; however, differences were not significant compared with placebo treatment (p = 0.113 and p = 0.558 for quarterly and monthly fremanezumab use vs. placebo, respectively). In the FOCUS trial that evaluated fremanezumab efficacy in patients with documented failure to up to four migraine preventive medication classes, the monthly fremanezumab group reported significantly improved PHQ-9 scores versus placebo (p = 0·0037) (Ferrari et al., 2019). The trials examining the effects of fremanezumab in patients with episodic migraine did not provide data on the average changes in PHQ-9, HDRS, or BDI-II scores.

### 3.2 Anxiety assessment using the GAD-7 and HARS tools

The primary clinical trials evaluating CGRP antagonists did not present assessments of anxiety using mean changes in GAD-7 or HARS scores. The single exception regarded the results from the CONQUER study that investigated galcanezumab efficacy in adults with treatment-resistant migraine. The study showed that the percentage of patients with possible anxiety disorder was decreased from 13.8 to 8.5 %, while the placebo group saw a change from 15.7 to 14.4 % (Maizels et al., 2020). Patients treated with galcanezumab- showed a numerically greater mean decrease in GAD-7 scores (−0.9 vs. − 0.4), but the difference did not reach statistical significance (p = 0.069) (Maizels et al., 2020).

Further, an RWE study of galcanezumab showed that anxiety, assessed using HARS, was significantly improved from baseline after the third and the sixth rounds of administration (from 13 ± 9 to 11.5 ± 7.5 and 10 ± 9, respectively; p < 0.002) (Silvestro et al., 2022).. Another RWE study of erenumab showed that anxiety, also assessed using HARS, was improved from baseline after the third and sixth rounds of erenumab administration, achieving statistical significance only after the sixth dose (from 17.1 ± 1.2 to 15.1 ± 1.7 and 13.2 ± 1.6, respectively; p < 0.05 for the sixth dose) (Russo et al., 2020).

## 4 Discussion

Many international guidelines and consensus statements provide recommendations on using monoclonal antibodies targeting the CGRP pathway for migraine prevention (Ailani, Burch, Robbins, & Society, 2021; Mitsikostas et al., 2023, Sacco et al., 2022). Nevertheless, not all of them have addressed the simultaneous presence of depression and anxiety in migraine patients. Various validated questionnaires have been developed to detect and assess depression and anxiety in patients with migraine. These include PHQ-9, HDRS, BDI-II, HARS, and GAD-7 (Kroenke et al., 2001, Bobo et al., 2016, Wang and Gorenstein, 2013, Spitzer et al., 2006, Seo and Park, 2015a, Maier et al., 1988; Park et al., 2020; Seo and Park, 2015b, O’Sullivan et al., 1997, Sharp, 2015).The literature extensively documents the validity and specificity of these questionnaires within the context of migraines. Moreover, many patient-reported validated questionnaires used in primary trials regarding treatment with CGRP antagonists have assessed the effect of migraines on productivity and quality of life, indirectly capturing the patient's depressive or anxiety state. However, the primary objective of administering these questionnaires was to measure the effect of migraines on daily activities. Although questionnaires such as the Migraine Disability Assessment (MIDAS), Headache Impact Test (HIT-6), and Migraine-Specific Quality of Life Questionnaire (MSQ) have reflected positive results in primary clinical trials evaluating most CGRP antagonists, direct speculations regarding improvement in concurrent depression or anxiety are not possible. In this review, our goal was to identify potential associations between the use of injectable CGRP antagonists and improvement in depression and anxiety by analyzing the results of validated questionnaires that assessed these comorbid conditions.

The findings indicate that the use of CGRP antagonists, specifically erenumab and galcanezumab, is linked to a significant decrease in mean PHQ-9 scores, suggesting a potential additional advantage in managing depression induced by or associated with migraines. This notion gains further support from RWEs, which report both clinical and statistically significant improvements in concurrent depression when using erenumab and galcanezumab, as assessed using additional validated measures such as HDRS and BDI-II (Talbot et al., 2021, Russo et al., 2020, Maizels et al., 2020, Silvestro et al., 2022). Fremanezumab, too, achieved a significantly greater improvement in the mean PHQ-9 scores in patients with documented failure of up to four migraine preventive medication classes (Lipton et al., 2021, Ferrari et al., 2019). We did not identify any studies reporting on the effect of eptinezumab on concurrent depression.

The GAD-7 questionnaire is a dependable instrument to assess anxiety severity and is commonly administered in research and clinical settings to screen and track anxiety disorders (Spitzer et al., 2006, Seo and Park, 2015a). In adults with treatment-resistant migraine, improvements in GAD-7 scores were observed with the use of galcanezumab compared to placebo (Maizels et al., 2020), although statistical significance was not attained. Nevertheless, RWE studies demonstrate favorable HARS scores with the administration of galcanezumab and erenumab, with differences in these instances achieving statistical significance at different timeframes. The demonstrated effect of galcanezumab is consistent with the absence of depressive elements and anxiety at the third dose, offering a relatively faster improvement in psychiatric comorbidities (Silvestro et al., 2022). This benefit could be explained by the loading dose required at drug initiation to achieve a faster serum steady-state concentration. In a study designed along similar lines, erenumab showed a similar effect after the sixth dose (Russo et al., 2020). In that study, a 70-mg monthly dose of erenumab was administered up to the third dose. Subsequently, patients who experienced a substantial reduction (≤30 % MMDs) continued with the 70 mg dosage, whereas those who did not were shifted to the higher 140 mg monthly dose. We did not identify any studies evaluating the effects of eptinezumab or fremanezumab on concurrent anxiety.

## 5 Conclusions

The available data show that CGRP antagonists have validated evidence of efficacy in improving concurrent depressive and anxiety symptoms, which are often experienced by individuals with migraines. Specifically, trials and post-hoc analyses evaluating galcanezumab and erenumab showed evidence based on validated assessments of concurrent depression and anxiety. Sufficient evidence on the effect of eptinezumab on these mental health disorders frequently experienced by patients with migraine are not available at this point. Among the evidence discussed in this paper, the most validated assessments appear to include galcanezumab, with favorable outcomes in improving depression and anxiety in shorter time frames. Although the trials collected scores using tools such as PHQ-9, HDRS, HARS, and GAD-7, these were not the primary outcome measures, and the studies were not specifically designed to investigate the effects of medications on depression or anxiety.

This research received no external funding. All aspects of this study, including design, data collection, analysis, and manuscript preparation, were carried out using the authors' own resources.

## Declaration of competing interest

The authors declare that they have no known competing financial interests or personal relationships that could have appeared to influence the work reported in this paper.
